# The development of spontaneous facial responses to others’ emotions in infancy: An EMG study

**DOI:** 10.1038/s41598-017-17556-y

**Published:** 2017-12-13

**Authors:** Jakob Kaiser, Maria Magdalena Crespo-Llado, Chiara Turati, Elena Geangu

**Affiliations:** 1 Ludwig-Maximilians-Universitaet Muenchen, Muenchen, Germany; 2 Lancaster University, Lancaster, England; 3 Università degli Studi di Milano-Bicocca, Milan, Italy; 4 University of York, York, United Kingdom

## Abstract

Viewing facial expressions often evokes facial responses in the observer. These spontaneous facial reactions (SFRs) are believed to play an important role for social interactions. However, their developmental trajectory and the underlying neurocognitive mechanisms are still little understood. In the current study, 4- and 7-month old infants were presented with facial expressions of happiness, anger, and fear. Electromyography (EMG) was used to measure activation in muscles relevant for forming these expressions: zygomaticus major (smiling), corrugator supercilii (frowning), and frontalis (forehead raising). The results indicated no selective activation of the facial muscles for the expressions in 4-month-old infants. For 7-month-old infants, evidence for selective facial reactions was found especially for happy (leading to increased zygomaticus major activation) and fearful faces (leading to increased frontalis activation), while angry faces did not show a clear differential response. These results suggest that emotional SFRs may be the result of complex neurocognitive mechanisms which lead to partial mimicry but are also likely to be influenced by evaluative processes. Such mechanisms seem to undergo important developments at least until the second half of the first year of life.

## Introduction

Emotional facial expressions are rich and powerful means of communicating information about one’s affective states, as well as about the environment in which we live in. Not surprisingly, by adulthood, we develop high expertise to process facial expressions fast and accurately. A testimony to their importance and saliency is the fact that the perception of emotional faces often elicits emotionally convergent facial responses in the observer. For example, during social interactions, we often respond rapidly with emotional facial expressions which are similar to those we observe in others, such as smiling when we see someone happy. These spontaneous facial responses (SFRs), which are sometimes covert and not visible through direct observation^1,2^, nonetheless are thought to play crucial roles in how we communicate and empathise with each other, as well as in establishing cohesive social groups^3,4^. Impairments in these social abilities are usually reported in pathologies characterised by atypical social functioning like autism, conduct disorders, and psychopathy^5,6^, and thus understanding the extent to which they are associated with atypical manifestations of emotional SFRs is of high importance. The study of infants’ spontaneous facial responses to others’ emotions is essential in this respect. Infancy is a crucial time period for tuning and optimising the brain circuitry for processing stimuli with socio-emotional relevance, setting the stage for both the refinement of the early acquired social skills and the emergence of new and more complex ones later in life^7–9^. In addition, infancy also provides unique opportunities for studying the SFRs to others’ emotions in relative isolation from the influence of cultural norms and values, as well as symbolic linguistic processing of emotional information. Despite their relevance, the systematic investigation of infants’ facial responses to others’ emotions is limited^10–12^. In order to address this developmental gap, in this study we investigated SFRs to dynamic facial expressions of emotions in 4- and 7-months-old infants using electromyography (EMG).

Different neurocognitive mechanisms have been proposed to underlie the SFRs which are congruent with others’ emotional expressions. One view regards them as instances of motor mirroring or motor mimicry, where the observation of others’ facial movements elicits the selective activation of the corresponding muscles in the observer. These responses are thought to be largely automatic, occurring outside the mimicker’s awareness, intention, and control^13,14^. In light of these characteristics, Chartrand and Bargh^15^ metaphorically referred to motor mirroring as the ‘chameleon effect’. Motor mimicry relies on perception-action matching mechanisms involving the shared representation of the observed and executed facial actions. At the neural level, the mirroring properties of a cortical network including the inferior frontal, premotor and inferior parietal cortex (mirror neuron system - MNS) are thought to be involved in implementing the perceived emotional facial expression onto observer’s own motor representations of producing that expression^16–18^. The simple sensory input of observing another’s action leads to an activation of an internal motor representation in the observer due to the similarity of the perceived action and the motor representation used to control action execution^19,20^. The relation between the motor cortex activation and the selective excitability of the muscles involved in performing an action has been regarded as supportive of this view^21^. The re-enactment of the observed expression could, in turn, even lead to the alteration of the observer’s own affective state through muscular feedback^15,22^. Indeed, numerous studies have shown that adults and older children rapidly mimic the facial expressions displayed by the people with whom they interact^23–25^.

However, several findings are difficult to integrate with this perception-action matching proposal. SFRs which seem to match the observed emotions have also been recorded in response to emotional cues other than faces (i.e., body postures, vocal expressions, arousing pictures^26–30^, thus in the absence of the corresponding motor model which is important for a simple perception-action matching account. Moreover, observing others’ facial expressions does not always elicit matching SFRs in the observer. For example, observing others’ angry faces elicits SFRs specific for fear rather than anger^25,31,32^. Angry individuals represent potential sources of threat^33,34^, and usually elicit fear in others, both at subjective and psychophysiological level^35,36^. Only when angry individuals are perceived as physically weaker and threatening one’s social status, their facial displays of anger elicit similar SFRs in the observer^26,37^. Situations of competition were also shown to trigger facial responses which are incongruent with the observed emotional expressions. Instead of showing positive emotional facial expressions, adults respond with negative displays to their competitors’ pleasure^38,39^. In all these examples, the facial responses converge with the meaning and the informative value for the observer of the emotional signals received from others, rather than their motor characteristics. Studies have also shown that posing a certain emotional expression can alter one’s subjective emotional experience^40–43^. However, the causal link between emotional facial mimicry and changes in affective state lacks definitive evidence^44^.

To account for these additional findings, it has been proposed that the SFRs which converge with the displays of affect observed in others involve emotion communicative processes^44–47^. At the heart of this emotion-communicative proposal is the idea that the evaluation of the information provided by the emotional cues for self is critical and varies as function of stimulus features and social context. The evaluation of the emotional information can occur at different levels, from relevance detection and coding the negative and positive reward value of the stimuli, to fast or more elaborate cognitive appraisal^48^. At the neural level, the evaluation of the emotional cues involves a circuitry consistent of both subcortical and cortical structures^48–51^, amongst which the amygdala, the brainstem, and the orbitofrontal cortex (OFC) have been extensively investigated (see Koelsch *et al*.^48^ for a recent review). For example, the amygdala plays a role in the fast detection and evaluation of threat^52–55^, as well as in the processing of happy events^56^. The amygdala shows connectivity and co-activation with the motor and pre-motor cortical structures involved in preparation for action^57–60^, suggesting that the early evaluation of emotional cues informs the behavioural responses during social interactions^45^. The shared motor representations comprising components of the perceived action and associated predicted somatosensory consequences are also considered to be active during the perception of emotional displays. However, the attributed role has more to do with the anticipation of others’ behaviour and intentions^44,61,62^. Components of the neural network underlying these processes are also thought to play a role in implementing the appropriate motor responses afforded by the specific social situation^62^. Recent neuroimaging investigations have shown that although the threat evaluation processes related to the amygdala slightly precede those involved in generating shared representations, these seem to interact and be integrated as soon as 200 ms after stimulus onset^63^. Given the role of the amygdala in evaluating a range of emotional events, a similar sequence of operations may also be encountered for positive emotions or for other brain structures with evaluative properties (e.g., the OFC) which are functionally connected with the motor cortex^48,51,56^.

In order to understand the factors that influence facial reactions, it is important to investigate the development of the infant SFRs to others’ emotional facial expressions. Recently it was shown that 5-months-old infants selectively respond with increased activation of the zygomaticus major to audio-visual recordings of adults smiling and with increased activation of the corrugator supercilli to audio-visual recordings of adults crying. This selective muscle activation was not reported for unimodal presentations of adult expressions of cry and laughter (i.e., voice-only, face-only)^12^. Nonetheless, the absence of angry expressions and of contrasts between different negative emotional expressions, together with the lack of a truly developmental perspective given that only one age group was tested, highly limit the conclusions that can be drawn based on these findings.

In the current study we employed an EMG paradigm which contrasts the responses towards three dynamic facial expressions of emotion (i.e., happiness, anger, and fear) in three facial muscles that have been found to be selectively activated in these facial displays (i.e., zygomaticus major for smiling during happiness, corrugator supercilli for frowning in anger, and frontalis for forehead raising in anger displays). The study was conducted with both 4- and 7-months-old infants. The choice of these age groups was motivated by the evidence suggesting that they represent important hallmarks in the development of the ability to process emotional information from faces^64^. Although even very young infants are able to discriminate between different facial expressions of emotions^65–67^, it seems that only beginning with the age of 7-months they rely on adults’ specific emotional expressions to guide their behaviour towards the stimuli in the environment^64,68,69^. For example, it is around this age that infants begin to perceive fearful facial expressions as specific cues for threat^64,68^.

If SFRs were predominantly a case of automatic perception-action matching, one would expect stronger activation in the muscle mainly involved in this expression (zygomaticus major for happy faces, corrugator supercilii for angry faces, and frontalis for fearful faces) relative to the other facial muscles. Cases where SFRs do not match facial expressions would support the view that additional mechanism to the direct mirror matching are responsible for SFRs, such as evaluative-communicative processes. From this perspective, emotion congruent SFRs are expected to occur at the age when infants are able to process the informative value of the perceived expression. In light of evidence suggesting that only towards the age of 7-months infants are more likely to process the informative value of certain emotional facial expressions, we anticipate SFRs congruent with the observed ones in 7- rather than 4-months-old infants. The comparisons across multiple emotions and multiple facial muscles at two developmental periods will allow us to draw conclusions with regard to the specificity and selectivity of the infant emotional SFRs.

## Results

Mean amplitude values expressed as z-scores were analysed using a mixed ANOVA with Muscle (frontalis, corrugator supercilli, zygomaticus major), Emotion (happy, anger, fear), and Time window (Time 1, Time 2) as within factors and Age group (4-months-old vs. 7-months-old) as a between factor. All statistical tests were conducted at the 0.05 level of significance (two-tailed), with Bonferroni correction for post-hoc comparisons. The results show significant interactions between Time window × Age group (*F*(1,49) = 5.466, *p* = 0.024, ${\eta }_{p}^{2}$ = 0.100), Emotion × Muscle × Age group (*F*(4,196) = 3.276, *p* = 0.013, ${\eta }_{p}^{2}$ = 0.063), as well as Emotion × Muscle × Time window × Age group (*F*(4,196) = 2.749, *p* = 0.029, ${\eta }_{p}^{2}$ = 0.053). No other significant main effects or interactions were observed (p > 0.052). Furthermore, to explore the Muscle × Emotion × Age Group × Time window interaction, we proceed to perform a 3 (Muscle: frontalis, corrugator or zygomaticus) × 3 (Emotion: happy, anger or fear) × 2 (Time window: Time 1, Time 2) repeated measures ANOVAs for each age group. Also, since we transformed facial reactions to z-scores, we were able to analyse whether the reactions to each emotion differed between muscles.

### 4-months-old infants

**Figure 1:**
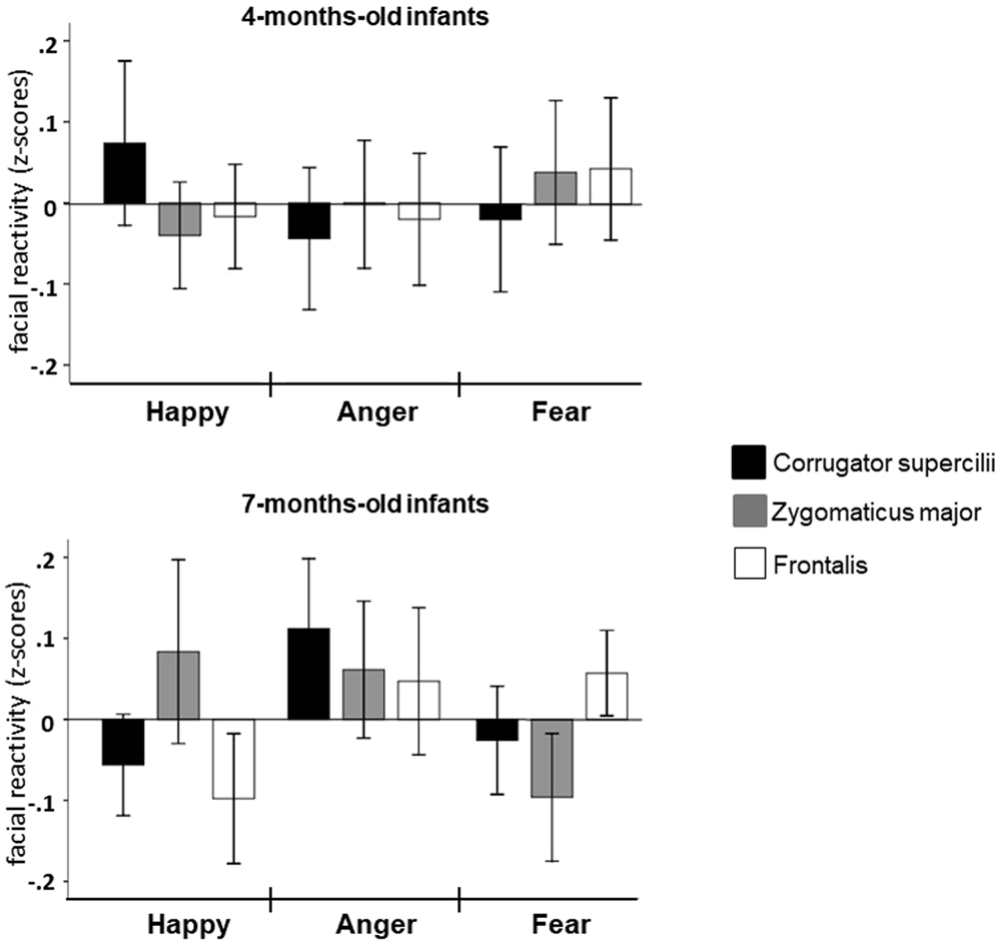
Means (and 95% confidence interval) of facial reactions towards the stimuli during Time 2 (1000–3000 ms from onset) for different muscles (expressed as z-scores).

For the 4-months-old group an ANOVA with the factors Emotion, Muscle, and Time window revealed a significant interaction Emotion × Muscle, *F*(4,104) = 3.275, *p* = 0.014, ${\eta }_{p}^{2}$ = 0.112 (Fig. 1). The post-hoc pairwise comparisons did not result in any significant differences in the muscle activation between emotions (*p* > 0.261), nor any differences in activation between muscles within emotions (*p* > 0.054). No other main effects or interactions were observed (*p* > 0.088). Thus we found no evidence of SFRs in the younger age group.

### 7-months-old infants

For the 7-month olds, the results show a significant interaction between Emotion, Muscle, and Time window, *F*(4,92) = 3.451; *p* = 0.011; ${\eta }_{p}^{2}$ = 0.130. No other main effects or interactions were observed (*p* > 0.052). This indicated that 7-months olds showed differential facial responses towards the emotional faces which are dependent on time. We conducted post-hoc pairwise comparisons in order to compare the effect of different emotions on each muscle. For the 0 to 1000 ms time window, no significant differences between emotions were found for any of the muscles (*p* > 0.213). For the 1000 to 3000 ms time window, the corrugator supercillii showed significantly stronger reactions towards angry faces (*M* = 0.112, *SE* = 0.042) than happy faces (*M* = −0.056, *SE* = 0.030), *p* = 0.042. There were no significant differences between angry and fearful faces (*M* = −0.026, *SE* = 0.032), *p* = 0.167, or between happy and fearful faces, *p* > 0.900. For the frontalis, we found significantly stronger activation for fearful (*M* = 0.057, *SE* = 0.026) than for happy faces (*M* = −0.098, *SE* = 0.039), *p* = 0.023. No significant differences were found between fearful and angry faces (*M* = 0.047, *SE* = 0.044), *p* > 0.900, or between angry and happy faces, *p =* 0.213. For the zygomaticus, no significant differences emerged between the emotion categories (all p-values > 0.074; Fig. 1). For the 0 to 1000 ms time window, no significant differences in activation between muscles were found for any emotional facial expression (*p* > 0.849). For the 1000 to 3000 ms time interval, happy facial expressions elicited higher zygomaticus major activation (*M* = 0.084, *SE* = 0.055) compared to the corrugator supercilii (*M* = −0.056, *SE* = 0.030), *p* = 0.036, and the frontalis (*M* = −0.098, *SE* = 0.039), *p* = 0.018. There was no significant difference in reaction towards happy faces between corrugator and frontalis, *p* = 0.783. For fearful faces, the frontalis (*M* = 0.057, *SE* = 0.026) showed a significantly higher activation than the zygomaticus (*M* = −0.096, *SE* = 0.038), *p* = 0.009. There was no significant difference for fearful faces between frontalis and corrugator supercilii (*M* = −0.026, *SE* = 0.032), *p* = 0.114, and no significant difference between corrugator supercilii and zygomaticus major, *p* = 0.316. For angry faces, no significant differences emerged between the muscles (all p-values > 0.849; Fig. 1).

## Discussion

Our aim was to understand the ontogeny of infants’ facial responsivity to others’ emotions and how this relates to the current theoretical models regarding the role of perception-action matching mechanisms and affect processes. We therefore presented 4- and 7-months-old infants with dynamic facial expressions of happiness, fear, and anger, while we used EMG to measure the activation of the muscles specific for expressing these emotions (i.e., zygomaticus major, frontalis, and corrugator supercilli, respectively). The results show that infants’ SFRs to dynamic emotional facial expressions undergo significant developmental changes towards the age of 7-months.

The 4-months-old infants in our study did not manifest selective activation of the recorded facial muscles in response to dynamic facial cues of emotions. In fact, as Fig. 1 shows, very little facial responsivity was present for this age group. These findings are in line with previous EMG studies which show that 5-months-old infants do not match their SFRs with dynamic facial expressions of cry and laughter without additional emotion-relevant auditory cues^12^, as well as a series of behavioural studies which reported a lack of selective emotional facial responsivity for 2–3-months-old infants and newborns^10,70^.

Our study shows for the first time that dynamic emotional facial expressions elicit selective SFRs in 7-month-old infants. Importantly, this pattern of responsivity was not generalizable across all emotional expressions. The comparisons of muscle activation between and within each emotion show that observing dynamic facial expressions of happiness leads to increased activation of the muscle specific for expressing this emotion (i.e., zygomaticus major) and decreased activation of the muscle involved in expressing fear (i.e., frontalis) and anger (i.e., corrugator supercilli). A similar pattern of selective SFRs was also recorded for fearful faces, with an increased activation of the frontalis and decreased activation of the muscle specific for expressing happiness (i.e., zygomaticus major). In contrast, the perception of angry faces tended to lead to a more non-differentiated pattern of facial responsivity. While the muscle specific for expressing anger, corrugator supercilli, did record an increased activation in response to angry faces compared to the happy ones, this was not associated with a decrease in the activation of the muscle specific for smiling (i.e., the zygomaticus major) nor the muscle specific for fear (i.e., the frontalis). Similar partial selectivity of the behaviourally coded facial responsivity has been previously reported in studies with 2- to 3-months-old and 6-months-old infants, in which responses to more than two emotional expressions during ecological mother-infant interactions were contrasted^71–73^.

Amongst the most prominent theoretical proposals for the neurocognitive mechanisms underlying the SFRs to others’ emotions are those attributing a primary role to perception-action matching mechanisms^16,18,22^. The fact that 7-months-old infants do not respond to all emotional expressions included in this study with matching SFRs in a selective manner suggests that these are less likely to be simple re-enactments of the observed expressions based on perception-action matching mechanisms. Another possibility might be that infants are only capable of showing matching SFRs after sufficient familiarity with certain expressions through prior exposure. However, it seems less likely that these results are due to differences in exposure to angry facial expressions. From around the age of 2-months, infants are exposed to parents’ facial expressions of anger. Although these are not as frequent as facial expressions of happiness^74^, they are probably as frequent as those of fear^75^, for which infants show congruent SFRs. Additionally, our findings are not likely to be due to an inability to perceptually discriminate or display the expressions tested. In particular, at this age infants have the ability to perceptually discriminate angry faces from various other emotional facial expressions^64,76^, as well as the ability to display the facial movements specific for anger, happiness and fearfulness^71,72,77–79^.

Behavioural and neuroimaging studies have shown that the more elaborate representations of emotional expressions and their communicative value develop in infants after the age of 5-months, in an emotion dependent fashion^64,76^. For example, 6–7-months-old but not younger infants show specific sensitivity to fearful faces as cues for threat and manifest increased attention towards objects that were looked at by fearful faces^64,68^. This ability consolidates in the next months^80^ and becomes more obvious in how infants interact with their environment around the age of 12-months^69,81,82^. Although emotional expressions of anger are also relevant cues for threat, infants do not seem sensitive to their specific informative value until closer to their first birthday^83,84^. The insufficiently developed ability of 7-month-old infants to evaluate the specific informative value of angry facial expressions may partially explain their lack of selective SFRs for this expression. The immature ability of the 4-months-olds to process a variety of facial expressions may also be partially responsible for the absence of selective SFRs across all expressions included in this study. Taken together, the age differences and pattern of selective muscle activation appear to be consistent with proposals that see SFRs not as pure motor mimicry, but also see the influence of communicative processes involving the evaluation of the emotional cues^11,26,44,45,72^.

This interpretation does not necessarily mean that instances of emotionally convergent SFRs may only be recorded in infants closer to the age of 7-months, but rather that they may be limited to those situations where infants are able to extract salient information from the perceived emotional cues. Previous behavioural studies which used more ecological adult-infant interaction paradigms showed that infants as young as 2–3-months manifest facial responses which tend to converge emotionally with the observed ones. However, these responses are specific to situations involving interactions between infants and their mothers, with whom they have had extensive experience in social exchanges^10,71–73,85^. In this case, infants’ facial responses may reflect the appraisal of the perceived emotional cues with respect to the mother’s immediate future actions which in the past elicited specific emotional responses. For example, caregivers’ smiling faces are typically associated with pleasant social engagement, such as play and caring actions known to induce positive affect in the infant. In contrast, the display of negative emotional expressions is more likely to be followed by a lack of social interaction which can be distressing for the infant^71,72,76^. This explanation would also account for those situations where the perception and the evaluation of others’ emotions are facilitated by the presence of multiple cues^26,27,86–89^ or the quality of the emotional cues (e.g., static versus dynamic expressions^86,90–92^). The fact that 5-month-old infants respond with emotion convergent SFRs to audio-visual expressions of laughter and crying but not to the unimodal presentations (i.e., face-only, voice-only) of these emotional displays^12^ may reflect such facilitating effect^93–95^.

Although the current findings together with those previously reported^10–12^ are informative about the emergence of the emotion congruent SFRs in infancy and suggestive with regards to the complexity of the underlying neurocognitive mechanisms, further research is needed in order to draw firmer conclusions in this respect. For example, although the current study shows that facial EMG paradigms can be successfully used with infants of different ages, it does not allow establishing whether the observed facial responses are related to changes in autonomic arousal. Emotional expressions displayed by both adults and peers were found to elicit autonomic arousal indicative of emotional responsivity in infants. In particular, changes in skin conductance and pupil diameter have been reported in response to expressions of happiness, fear, anger, and general distress in infants starting with the age of 4-months^96–99^. Changes in autonomic arousal also seem to be significantly related to infants’ facial responses in emotion elicitation situations^100–103^. Thus, concurrent facial EMG and measures of psychophysiological arousal would be particularly valuable for understanding how affect related processes contribute to the emergence of the emotionally convergent SFRs during infancy and childhood. Such knowledge is also directly relevant for studying the ontogeny of affect sharing and empathy^104,105^.

Extracting, processing, and responding to the emotional information presented by human faces relies on complex neural networks involving both sub-cortical and cortical structures, including those that are part of the emotion-related brain circuits (e.g., the amygdala and the orbito-frontal cortex^49,50,106^) and those functionally linked with motor preparation for action and estimating others’ immediate intent for action^45,57,59,60,62,107–109^. Although the emotion-related brain structures are already functional at birth, and the connections with the other related cortical and subcortical areas established, these brain structures continue to mature and their pattern of connectivity refines over the course of postnatal development^75^. It is thus possible that the SFRs of the 7-months-old infants to happy and fearful facial expressions reflect, at least partially, these developmental changes in the underlying neural network^75,110^. Natural variations in the familiarity with different social contexts, as well as in the maturation of the relevant brain networks which are specific to the first year of life can thus provide unique opportunities for characterizing processes that would otherwise be impossible to capture in the fully mature adults^111,112^.

Different experimental approaches could be adopted for further investigations into the neurocognitive mechanisms underlying emotion congruent SFRs in infancy. For example, concurrent recordings of facial EMG and EEG based measures of cortical activation would be particularly informative in understanding how neural development contributes to the emergence of emotionally convergent SFRs in infancy^44,62,112^. These paradigms have the potential to clarify the extent to which shared motor representations comprising components of the perceived action and associated somatosensory consequences are involved in generating emotion congruent SFRs in infants, alongside emotion evaluation and reactivity processes. Specifying the dynamic of the facial muscle activation may also be relevant in this respect, potentially reflecting the chronology of different processes. In the present study we have shown that the selective facial muscle activation specific for emotion congruent SFRs is overall recorded between 1000 and 3000ms after stimulus onset. This timing is similar to that reported in previous studies with young children^32^. Nevertheless, more subtle latency differences between emotions, and between muscles within emotion categories, may be present^113^. The stimuli used in the current study were not matched for the precise timing of facial actions, therefore not allowing a more refined time sensitive analysis. Artificially developed stimuli, such as morphed faces, or static facial expressions would be particularly suitable in this respect.

Being able to detect and respond to others’ emotions is essential to our social lives. For the past decades, a large number of studies have shown that adults tend to respond with rapid facial responses which converge emotionally with the emotions they perceive in others^86^. Although much more limited, evidence also emerged in recent years to show that similar patterns of facial responsivity can be reported during childhood^31,114–116^. Despite being a well-documented phenomenon in adulthood, debates regarding its early ontogeny and the underlying neurocognitive mechanisms remain open^10,12,27,44^. Our study shows that spontaneous facial responses which converge emotionally with the facial expressions observed in others can be recorded in 7- but not in 4-months-old infants. The pattern of infant emotional SFRs suggests that they may rely on complex neurocognitive mechanisms^44^, which undergo important developments at least until the second half of the first year of life. The factors contributing to the development of infants’ emotional SFRs remain to be established, with direct relevance for understanding the emergence of related complex social abilities like communication and empathy^105,117^.

## Methods

### Participants

Twenty seven 4-month old infants (11 females, M_age_ = 135.11 days, SD = 10.08 days) and 24 7-month old infants (14 females, M_age_ = 226.17 days, SD = 9.90 days) were included in the final analysis. An additional 5 4-months-old and 8 7-months-old infants were tested but not included in the final sample due to technical issues (n = 4) or inattentiveness resulting in less than 5 good trials per condition (n = 10). All participants were recruited from a small urban area in North West England. Informed consent was obtained from all parents prior to the beginning of the procedure. The procedure was carried out in accordance with the ethical standards of the Declaration of Helsinki (BMJ 1991; 302:1194). Ethical approval was granted by the Lancaster University Ethics Committee. Parents were reimbursed for their travel expenses (£10), while infants received a token for their participation.

### Stimuli

Fifteen grey-scale dynamic female human faces displaying happiness (n = 5), anger (n = 5), and fear (n = 5) were taken from the Cohn-Kanade Expression database^118^, which has become one of the most widely used stimuli for studies of facial expression analysis^118,119^. One of the main strengths of this dataset is that all facial expressions have been fully FACS coded^119^. The chosen faces were selected for their emotional valence. The selection criteria for the stimuli was that all happy facial expressions included corners of the mouth raised in a smile, all anger expressions included furrowed brows, and all fear expressions included raised eyebrows. For all stimuli, the transition between neutral and emotional expression occurred between 0 and 1000 ms, while the peak expressivity was reached between 1000 and 3000 ms. The exact timing of the facial movements, specific for each emotion expression, varied within and between stimulus categories. Face images were cropped using an oval frame that allowed facial features to be visible but excluded hair, ears, and any other paraphernalia.

### Procedure

Participants were tested individually in a quiet and dimly lit room. Before placing the electrodes, the skin was cleaned with an alcohol-free wipe. The electrodes were attached by one of the experimenters, while the second blew soap bubbles or manipulated a rattle toy in order to maintain the participant calm and distract him/her, as needed. Once the facial electrodes were placed, the participants sat during the entire procedure on their mothers’ lap approximately 70 cm away from a 24-inch monitor. Parents were instructed to hold their infants’ hand as still as possible to prevent infants from pulling the facial electrodes, not to speak to them, and not to point towards the screen during the entire stimuli presentation.

**Figure 2:**
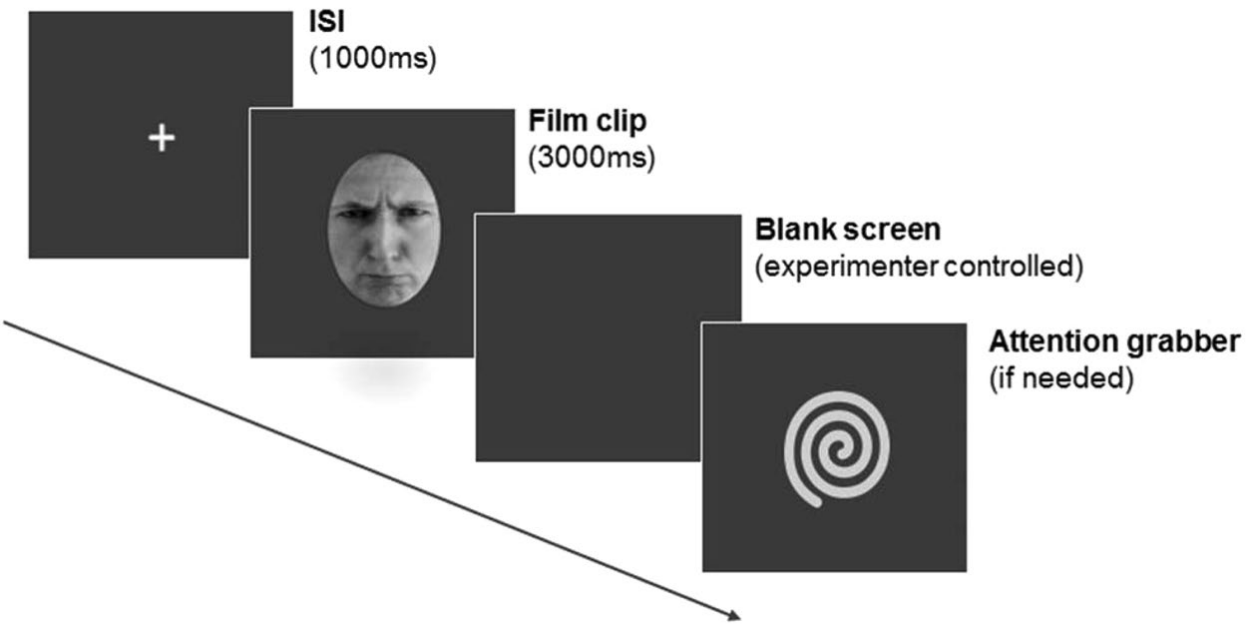
Example of a trial structure and stimuli used in the study. After a 1000 ms central fixation cross, the participants were presented for 3000 ms with the dynamic facial expression of either anger, happiness or fear displayed by a female adult. The emotional stimulus was followed up by a blank screen. The non-social attention grabber was presented whenever it was required to recapture participants’ attention to the screen. (The face picture included in the figure is for illustration purposes only and not part of the stimuli used in the study).

Each trial started with a central fixation cross for 1000 ms, during which baseline muscle activity levels were established. Following the fixation cross, a black screen displaying the emotional facial expression appeared for 3000 ms, followed by a blank screen (see Fig. 2). Between trials, a dynamic non-social attention grabber was played whenever needed in order to maintain the participants’ attention to the stimuli in case they showed signs of becoming distracted. The option of having experimental controlled presentation of the attention grabber rather than an automatic presentation after each trial is common to infant psychophysiology paradigms requiring the presentation of many trials^120^ and capitalizes on the infants’ natural bouts of attention. The procedure continued for as long as infants paid attention to the stimuli. On average, participants completed 55.12 trials (Happy faces: *M* = 18.35 trials, *Min* = 10, *Max* = 30; Angry faces: *M* = 18.12 trials, *Min* = 10, *Max* = 30; Fearful faces: *M* = 18.65 trials, *Min* = 11, *Max* = 30). The entire procedure was video recorded in order to establish whether the infants had watched the faces in each trial and to facilitate artifact detection during the data analysis. The complete experimental session took approximately 10 min.

### EMG data acquisition and analysis

Electromyography was used to record the levels of muscle activity over the zygomaticus major (raises the cheek), the medial frontalis (raises the brow), and the corrugator supercilli (knits brow). This method was extensively used to record adults’ facial responses to others’ emotions^39^. Although the internal consistency of the recorded EMG signal in these studies tends to be low, the test-retest reliability is good^121^. Recent studies show that facial EMG is a method suitable to be used with young children and infants^31,32,122^. In the present study, a BIOPAC MP30 continuously recorded the EMG signal from the selected muscles using bipolar montages. Disposable surface adhesive 4 mm Ag-AgCl EMG electrodes (Unimed) were placed on the infants’ face at locations corresponding to each muscle according to the guidelines by Fridlund & Cacioppo^123^ and as previously reported in facial EMG studies with infants^122,124^ and toddlers^32^. Electrodes were positioned on the left side of the face to obtain maximal reactions^123^. The reference electrode was positioned just below the hairline approximately 3 cm above the nasion. The EMG signal was recorded at a sampling rate of 1 kHz filtered offline (low pass: 150 Hz; high pass: 30 Hz), and rectified. Rectified data was averaged in 200 ms time bins which where z-transformed for each muscle and participant individually. This is a standard procedure in facial EMG studies allowing for a comparison between participants and muscles (see Supplementary Information – Fig. 2 - for a depiction of the EMG signal before standardization). Participants’ looking time toward the screen was coded offline in order to inform whether they attended the stimuli. This is common procedure in electrophysiology research with preverbal children (e.g., Lloyd-Fox *et al*.^125^). Trials with a looking time of less than 70% of the stimulus duration, as well as trials with excessive movement or noise artifacts were excluded. Only children with minimum five trials per condition were included in the final statistical analyses. This criterion was informed by previous studies with infants^12^, children^31,114^, and adults^24,126–128^. Across participants, the mean number of trials contributing to the final statistical analyses was 33.10 (Happy faces: *M* = 11.04 trials, *Min* = 5, *Max* = 18; Angry faces: *M* = 10.18, *Min* = 5, *Max* = 17; Fearful faces: *M* = 11.88, *Min* = 5, *Max* = 19).

Previous studies with children using a similar paradigm suggest that facial reactions towards emotional expressions start to show between 500 and 1000 ms for static facial stimuli that are already fully developed in their expressivity^31,32,115^, which is also consistent with adult studies^23,25,128^. As the dynamic stimuli in this study gradually developed over the first 1000 ms and remained at peak between the 1000–3000 ms, we averaged for each trial both the first onset phase (Time point 1) and the peak expression phase (Time point 2). Average activation was baseline-corrected by subtracting the 1000 ms interval immediately before stimulus onset, and the mean across trials of the same emotion was calculated.

## Electronic supplementary material

Supplementary Information

## References

[1] Cacioppo JT, Petty RE, Losch ME, Kim HS (1986). Electromyographic activity over facial muscle regions can differentiate the valence and intensity of affective reactions. J. Pers. Soc. Psychol..

[2] Tassinary LG, Cacioppo JT (1992). Unobservable facial actions and emotion. Psychol. Sci..

[3] Hatfield E, Cacioppo JT, Rapson RL (1993). Emotional contagion. Curr. Dir. Psychol. Sci..

[4] Hess U, Fischer A (2013). Emotional mimicry as social regulation. Personal. Soc. Psychol. Rev..

[5] Lockwood PL, Bird G, Bridge M, Viding E (2013). Dissecting empathy: high levels of psychopathic and autistic traits are characterized by difficulties in different social information processing domains. Front. Hum. Neurosci..

[6] Bird G, Viding E (2014). The self to other model of empathy: Providing a new framework for understanding empathy impairments in psychopathy, autism, and alexithymia. Neurosci. Biobehav. Rev..

[7] Johnson MH (2011). Interactive Specialization: A domain-general framework for human functional brain development?. Dev. Cogn. Neurosci..

[8] Sirois S (2008). Précis of neuroconstructivism: how the brain constructs cognition. Behav. Brain Sci..

[9] Karmiloff-Smith A (1998). Development itself is the key to understanding developmental disorders. Trends Cogn. Sci..

[10] Oostenbroek J (2016). Comprehensive longitudinal study challenges the existence of neonatal imitation in humans comprehensive longitudinal study challenges the existence of neonatal imitation in humans. Curr. Biol..

[11] Haviland JM, Lelwica M (1987). The induced affect response: 10-week-old infants’ responses to three emotion expressions. Dev. Psychol..

[12] Isomura, T. & Nakano, T. Automatic facial mimicry in response to dynamic emotional stimuli in five-month-old infants. *Proc. R. Soc. B* (2016). 10.1098/rspb.2016.1948 PMC5204153 27974522

[13] Heyes C (2011). Automatic imitation. Psychol. Bull..

[14] Campbell, M. E. J. & Cunnington, R. More than an imitation game: Top-down modulation of the human mirror system. *Neurosci. Biobehav. Rev*., 10.1016/j.neubiorev.2017.01.035 (2017). 10.1016/j.neubiorev.2017.01.035 28153686

[15] Chartrand TL, Bargh JA (1999). The chameleon effect: the perception-behavior link and social interaction. J. Pers. Soc. Psychol..

[16] Carr L, Iacoboni M, Dubeau M-C, Mazziotta JC, Lenzi GL (2003). Neural mechanisms of empathy in humans: a relay from neural systems for imitation to limbic areas. Proc. Natl. Acad. Sci. USA.

[17] Dapretto, M. *et al*. Understanding emotions in others: mirror neuron dysfunction in children with autism spectrum disorders. *Nat. Neurosci.,* **9**, 2005–2007 (2006). 10.1038/nn1611 PMC3713227 16327784

[18] Pfeifer JH, Iacoboni M, Mazziotta JC, Dapretto M (2008). Mirroring others’ emotions relates to empathy and interpersonal competence in children. Neuroimage.

[19] Brass M, Heyes C (2005). Imitation: is cognitive neuroscience solving the correspondence problem?. Trends Cogn. Sci..

[20] Blakemore S-J, Frith CD (2005). The role of motor contagion in the prediction of action. Neuropsychologia.

[21] Fadiga, L., Fogassi, L., Pavesi, G. & Rizzolatti, G. Motor facilitation during action observation: A magnetic stimulation study. *J. Neurophysiol*. **73** (1995). 10.1152/jn.1995.73.6.2608 7666169

[22] Hatfield, E., Cacioppo, J. T. & Rapson, R. L. *Emotional contagion. Cambridge studies in emotion and social interaction*. (Cambridge University Press., 1994).

[23] Dimberg U (1982). Facial reactions to facial expressions. Psychophysiology.

[24] Dimberg U, Thunberg M, Elmehed K (2000). Unconscious facial reactions to emotional facial expressions. Psychol. Sci..

[25] Moody EJ, Mcintosh DN, Mann LJ, Weisser KR (2007). More Than Mere Mimicry?. The Influence of Emotion on Rapid Facial Reactions to Faces..

[26] Soussignan R (2013). Self-Relevance Appraisal of Gaze Direction and Dynamic Facial Expressions: Effects on Facial Electromyographic and Autonomic Reactions. Emotion.

[27] Magnée MJCM, de Gelder B, van Engeland H, Kemner C (2007). Facial electromyographic responses to emotional information from faces and voices in individuals with pervasive developmental disorder. J. Child Psychol. Psychiatry..

[28] Tamietto, M., de Gelder, B. & Tamietto, M, de Gelder, B. Emotional contagion for unseen bodily expressions: Evidence from facial EMG. *2008 8th IEEE Int*. *Conf. Autom. Face Gesture Recognit*. 1–5, 10.1109/AFGR.2008.4813317 (2008).

[29] Hietanen JK, Surakka V, Linnankoski I (1998). Facial electromyographic responses to vocal affect expressions. Psychophysiology.

[30] Dimberg U, Karlsson B (1997). Facial reactions to different emotionally relevant stimuli. Scand. J. Psychol..

[31] Beall PM, Moody EJ, Mcintosh DN, Hepburn SL, Reed CL (2008). Rapid facial reactions to emotional facial expressions in typically developing children and children with autism spectrum disorder. J. Exp. Child Psychol..

[32] Geangu E, Quadrelli E, Conte S, Croci E, Turati C (2016). Three-year-olds’ rapid facial electromyographic responses to emotional facial expressions and body postures. J. Exp. Child Psychol..

[33] Adams RBJ, Gordon HL, Baird AA, Ambady N, Kleck RE (2003). Effects of Gaze on Amygdala Sensitivity to Anger and Fear Faces. Science (80-.)..

[34] Frijda, N. H. *The emotions (Studies in emotion and social interaction)*. (Cambridge University Press, 1987).

[35] Davis, M. & Whalen, P. J. The amygdala: vigilance and emotion. *Mol. Cell* 13–34 (2001). 10.1038/sj.mp.4000812 11244481

[36] Critchley HD (2005). Activity in the human brain predicting differential heart rate responses to emotional facial expressions. Neuroimage.

[37] Hess U, Adams RB, Kleck RE (2005). Who may frown and who should smile? Dominance, affiliation, and the display of happiness and anger. Cogn. Emot..

[38] Lanzetta JT, Englis BG (1989). Expectations of Cooperation and Competition and Their Effects on Observers’ Vicarious Emotional Responses. J. Pers. Soc. Psychol..

[39] Likowski KU, Muhlberger A, Seibt B, Pauli P, Weyers P (2011). Processes Underlying Congruent and Incongruent Facial Reactions to Emotional Facial Expressions. Emotion.

[40] Bush LK, Barr CL, Mehugo GJ, Lanzetta JT (1989). The Effects of Facial Control and Facial Mimicry on Subjective Reactions to Comedy Routines 1. Motiv. Emot..

[41] Hennenlotter A (2009). The Link between Facial Feedback and Neural Activity within Central Circuitries of Emotion — New Insights from Botulinum Toxin-Induced Denervation of Frown Muscles. Cereb. Cortex.

[42] Soussignan RDS (2002). Emotional Experience, and Autonomic Reactivity. A Test of the Facial Feedback Hypothesis Duchenne Smile, Emotional Experience, and Autonomic Reactivity: A Test of the Facial Feedback Hypothesis. Emotion.

[43] Duclos SE (1989). Emotion specific effects of facial expressions on memory for life events Emotion-Specific Effects of Facial Expressions and Postures on Emotional Experience. J. Pers. Soc. Psychol..

[44] Dezecache, G., Eskenazi, T. & Grèzes, J. In *Shared Representations: Sensorimotor Foundations of Social Life* (eds Obhi, S. S. & Cross, E. S.) (Cambridge University Press, 2016).

[45] Grèzes, J. & Dezecache, G. How do shared-representations and emotional processes cooperate in response to social threat signals? *Neuropsychologia* 1–10, 10.1016/j.neuropsychologia.2013.09.019 (2014). 10.1016/j.neuropsychologia.2013.09.019 24080262

[46] Dezecache, G., Soussignan, R., Gre, J. & Conty, L. Self-relevance appraisal influences facial reactions to emotional body expressions. *Emotion* **8** (2013). 10.1371/journal.pone.0055885 PMC3566069 23405230

[47] Fischer AH, Hess U (2017). Mimicking emotions. Curr. Opin. Psychol..

[48] Koelsch S (2015). The quartet theory of human emotions: An integrative and neurofunctional model. Phys. Life Rev..

[49] Adolphs, R. Neural systems for recognizing emotion. *Curr. Opin. Neurobiol.*,169–177 (2002). 10.1016/s0959-4388(02)00301-x 12015233

[50] Vuilleumier, P., Armony, J. L., Driver, J. & Dolan, R. J. Distinct spatial frequency sensitivities for processing faces and emotional expressions. *Nat. Neur.*, **6**, 624–631 (2003). 10.1038/nn1057 12740580

[51] Pessoa L (2017). Opinion A Network Model of the Emotional Brain. Trends Cogn. Sci..

[52] Adolphs R (2010). What does the amygdala contribute to social cognition?. Ann. N. Y. Acad. Sci..

[53] Ledoux JE (1995). Emotion: Clues from the Brain. Annu. Rev. Psychol..

[54] Hoffman KL, Gothard KM, Schmid MC, Logothetis NK (2007). Facial-expression and gaze-selective responses in the monkey amygdala. Curr. Biol..

[55] Vuilleumier P, Richardson MP, Armony JL, Driver J, Dolan RJ (2004). Distant influences of amygdala lesion on visual cortical activation during emotional face processing. Nat. Neurosci..

[56] Sander D, Grafman J, Zalla T (2003). The human amygdala: an evolved system for relevance detection. Rev. Neurosci..

[57] Baumgartner T, Willi M, Jancke L (2007). Modulation of corticospinal activity by strong emotions evoked by pictures and classical music: a transcranial magnetic stimulation study. Neuroreport.

[58] Balconi M, Grippa E, Vanutelli ME (2015). What hemodynamic (fNIRS), electrophysiological (EEG) and autonomic integrated measures can tell us about emotional processing. Brain Cogn..

[59] Oliveri M (2003). Influence of the supplementary motor area on primary motor cortex excitability during movements triggered by neutral or emotionally unpleasant visual cues. Exp. Brain Res..

[60] Schutter DJLG, Hofman D, Honk JVAN (2008). Fearful faces selectively increase corticospinal motor tract excitability: A transcranial magnetic stimulation study. Psychophysiology.

[61] Kilner JM, Friston KJ, Frith CD (2007). Predictive coding: an account of the mirror neuron system. Cogn. Process..

[62] Hamilton, A. In *Shared Representations: Sensorimotor Foundations of Social Life* (eds Obhi, S. S. & Cross, E. S.) 313–331 (Cambridge University Press., 2016).

[63] Conty L, Dezecache G, Hugueville L, Grezes J (2012). Early Binding of Gaze, Gesture, and Emotion: Neural Time Course and Correlates. J. Neurosci..

[64] Hoehl, S. In *Children and Emotion. New Insights into Developmental Affective Sciences* (ed. Lagattuta, K. H.) 1–12, 10.1159/000354346 (2014).

[65] Farroni T, Menon E, Rigato S, Johnson MH (2007). The perception of facial expressions in newborns. Eur. J. Dev. Psychol..

[66] Serrano JM, Iglesias J, Loeches A (1995). Infants’ responses to adult static facial expressions. Infant Behav. Dev..

[67] Barrera ME, Maurer D (1981). The Perception of Facial Expressions by the Three-Month-Old. Child Dev..

[68] Hoehl S, Striano T (2010). The development of emotional face and eye gaze processing. Dev. Sci..

[69] Striano T, Vaish A (2006). Seven- to 9-month-old infants use facial expressions to interpret others’ actions. Br. J. Dev. Psychol..

[70] Kaitz M, Meschulach-sarfaty O, Auerbach J, Eidelman A (1988). A Reexamination of Newborns’ Ability to Imitate Facial Expressions. Dev. Psychol..

[71] Haviland JM, Lelwica M (1987). The induced affect response: 10-week-old infants’ responses to three emotion expressions. Dev. Psychol..

[72] Walker-Andrews AS (1997). Infants’ Perception of Expressive Behaviors: Differentiation of Multimodal Information. Psychol. Bull..

[73] Izard CE (1995). The Ontogeny and Significance of Infants’ Facial Expressions in the First 9 Months ofLife. Dev. Psychol..

[74] Malatesta ZC, Grigoryev P, Lamb C, Albin M, Culver C (1986). Emotion Socialization and Expressive Development in Preterm and Full-Term Infants. Child Dev..

[75] Leppänen JM, Nelson CA (2009). Tuning the developing brain to social signals of emotions. Nat. Rev. Neurosci..

[76] Grossmann T (2010). The development of emotion perception in face and voice during infancy. Restor. Neurol. Neurosci..

[77] Camras LA, Shutter JM (2010). Emotional facial expressions in infancy. Emot. Rev..

[78] Messinger D, Fogel A (2007). The Interactive Development Of Social Smiling. Adv. Child Dev. Behav..

[79] Messinger DS (2002). Positive and Negative: Infant Facial Expressions and Emotions. Curr. Dir. Psychol. Sci..

[80] Hoehl S, Reid V, Mooney J, Striano T (2008). What are you looking at? Infants’ neural processing of an adult’ s object-directed eye gaze. Dev. Sci..

[81] Campos JJ, Thein S, Owen D (2003). Human Infancy Emotional Expressions as Behavior Regulators. Ann. N. Y. Acad. Sci..

[82] Striano T, Rochat P (2000). Emergence of Selective Social Referencing in Infancy. Infancy.

[83] Grossmann T, Striano T, Friederici AD (2007). Developmental changes in infants’ processing of happy and angry facial expressions: a neurobehavioral study. Brain Cogn..

[84] Missana M, Grigutsch M, Grossmann T (2014). Developmental and Individual Differences in the Neural Processing of Dynamic Expressions of Pain and Anger. PLoS One.

[85] Bigelow AE, Rochat P (2006). Two-Month-Old Infants’ Sensitivity to Social Contingency in Mother-Infant and Stranger-Infant Interaction. Infancy.

[86] Seibt B, Mühlberger A, Likowski KU, Weyers P (2015). Facial mimicry in its social setting. Front. Psychol..

[87] Rychlowska, M., Zinner, L., Musca, S. C. & Niedenthal, P. M. From the eye to the heart: Eye contact triggers emotion simulation. In *Proceedings of the 4th Workshop on Eye Gaze in Intelligent Human Machine Interaction* **5** (2012).

[88] Magnee MJCM, Stekelenburg JJ, Kemner C, Gelder BD (2007). Similar facial electromyographic responses to faces, voices, and body expressions. Neuroreport.

[89] Schrammel F, Pannasch S, Graupner S-T, Mojzisch A, Velichkovsky BM (2009). Virtual friend or threat? The effects of facial expression and gaze interaction on psychophysiological responses and emotional experience. Psychophysiology.

[90] Rymarczyk K, Biele C, Grabowska A, Majczynski H (2011). EMG activity in response to static and dynamic facial expressions. Int. J. Psychophysiol..

[91] Sato W, Yoshikawa S (2007). Spontaneous facial mimicry in response to dynamic facial expressions. Cognition.

[92] Weyers P, Muhlberger A, Hefele C, Pauli P (2006). Electromyographic responses to static and dynamic avatar emotional facial expressions. Psychophysiology.

[93] Vaillant-Molina M, Bahrick LE, Flom R (2013). Young Infants Match Facial and Vocal Emotional Expressions of Other Infants. Infancy.

[94] Burnham D (1993). Visual recognition of mother by young infants: facilitation by speech. Perception.

[95] Flom R, Bahrick LE (2007). The Development of Infant Discrimination of Affect in Multimodal and Unimodal Stimulation: The Role of Intersensory Redundancy. Dev. Psychol..

[96] Geangu E, Hauf P, Bhardwaj R, Bentz W (2011). Infant pupil diameter changes in response to others’ positive and negative emotions. PLoS One.

[97] Gredebäck G, Eriksson M, Schmitow C, Laeng B, Stenberg G (2012). Individual differences in face processing: Infants’ scanning patterns and pupil dilations are influenced by the distribution of parental leave. Infancy.

[98] Jessen S, Altvater-Mackensen N, Grossmann T (2016). Pupillary responses reveal infants’ discrimination of facial emotions independent of conscious perception. Cognition.

[99] Nava E, Romano D, Grassi M, Turati C (2016). Skin conductance reveals the early development of the unconscious processing of emotions. Cortex.

[100] Emde RN, Campos J (1978). Infant Smiling at Five and Nine Months: Analysis of Heartrate and Movement. Infant Behav. Dev..

[101] Mattson WI (2013). Emotional expression and heart rate in high-risk infants during the face-to-face/still-face. Infant Behav. Dev..

[102] Lewis, M., Ramsay, D. S. & Sullivan, M. W. The relation of ANS and HPA activation to infant anger and sadness response to goal blockage. *Dev. Psychobiol*. 397–405, 10.1002/dev (2006). 10.1002/dev.20151 PMC1482732 16770761

[103] Lewis M, Ramsay D (2005). Infant Emotional and Cortisol Responses to Goal Blockage. Child Dev..

[104] Decety J, Michalska KJ (2010). Neurodevelopmental changes in the circuits underlying empathy and sympathy from childhood to adulthood. Dev. Sci..

[105] Decety J (2015). The neural pathways, development and functions of empathy. Curr. Opin. Behav. Sci..

[106] Pessoa L (2008). On the relationship between emotion and cognition. Nat. Rev. Neurosci..

[107] Balconi M, Bortolotti A (2013). Brain and Cognition The ‘“ simulation”’ of the facial expression of emotions in case of short and long stimulus duration. The effect of pre-motor cortex inhibition by rTMS. Brain Cogn..

[108] Coombes SA, Tandonnet C, Cauraugh JH, Summers JJ (2009). Emotion and motor preparation: A transcranial magnetic stimulation study of corticospinal motor tract excitability. Cogn. Affect. Behav. Neurosci..

[109] Coelho CM, Lipp OV, Marinovic W, Wallis G, Riek S (2010). Neuroscience Letters Increased corticospinal excitability induced by unpleasant visual stimuli. Neurosci. Lett..

[110] Leppanen JM, Nelson CA (2012). Early Development of Fear Processing. Curr. Dir. Psychol. Sci..

[111] De Haan M, Humphreys K, Johnson MH (2002). Developing a brain specialized for face perception: A converging methods approach. Dev. Psychobiol..

[112] Filippi CA (2016). Motor System Activation Predicts Goal Imitation in 7-Month-Old Infants. Psychol. Sci..

[113] Achaibou, A., Pourtois, G., Schwartz, S. & Vuilleumier, P. Simultaneous recording of EEG and facial muscle reactions during spontaneous emotional mimicry. *Neuropsychologia* 1–10, 10.1016/j.neuropsychologia.2007.10.019 (2007). 10.1016/j.neuropsychologia.2007.10.019 18068737

[114] de Wied M, van Boxtel A, Zaalberg R, Goudena PP, Matthys W (2006). Facial EMG responses to dynamic emotional facial expressions in boys with disruptive behavior disorders. J. Psychiatr. Res..

[115] Oberman LM, Winkielman P, Ramachandran VS (2009). Slow echo: facial EMG evidence for the delay of spontaneous, but not voluntary, emotional mimicry in children with autism spectrum disorders. Dev. Sci..

[116] Deschamps PKH, Coppes L, Kenemans JL, Schutter DJLG, Matthys W (2015). Electromyographic Responses to Emotional Facial Expressions in 6–7 Year Olds with Autism Spectrum Disorders. J. Autism Dev. Disord..

[117] Geangu, E. In *International Encyclopedia of the Social and Behavioral Sciences* (ed. Wright, J. D.) **7**, 549–553 (Elsevier, 2015).

[118] Kanade, T. & Cohn, J. F. Comprehensive database for facial expression analysis. In *Fourth IEEE International Conference of The Robotics Institute,* 46–53 (2000).

[119] Lucey, P. *et al*. The extended Cohn-Kanade Dataset (CK+): A complete dataset for action unit and emotion-specified expression. In *Computer Vision and Pattern Recognition Workshops (CVPRW), IEEE Computer Society Conference* 94–101 (2010).

[120] de Haan, M. *Infant EEG and event-related potentials*. (Psychology Press, 2013).

[121] Hess, U. *et al*. Reliability of surface facial electromyography. **54**, 12–23 (2017). 10.1111/psyp.12676 28000263

[122] Turati C (2013). The early development of human mirror mechanisms: evidence from electromyographic recordings at 3 and 6 months. Dev. Sci..

[123] Fridlund A, Cacioppo JT (1986). Guidelines for Human Electromyographic Research. Psychophysiology.

[124] Natale E (2014). Predicting others’ intention involves motor resonance: EMG evidence from 6- and 9-month-old infants. Dev. Cogn. Neurosci..

[125] Lloyd-fox S (2017). Cortical specialisation to social stimuli from the first days to the second year of life: A rural Gambian cohort. Dev. Cogn. Neurosci..

[126] Hess U, Blairy S (2001). Facial mimicry and emotional contagion to dynamic emotional facial expressions and their influence on decoding accuracy. Int. J. Psychophysiol..

[127] Hess U, Philippot P, Blairy S, Hess U, Philippot P (1998). Facial Reactions to Emotional Facial Expressions: Affect or Cognition? Facial Reaction s to Em otion al Facial Exp ressions: Affect or Cogn ition?. Cogn. Emot..

[128] Dimberg ULF, Petterson M (2000). Facial reactions to happy and angry facial expressions: Evidence for right hemisphere dominance. Psychophysiology.

