# Supplementary material for: The development of spontaneous facial responses to others’ emotions in infancy: An EMG study
Source: Sci Rep. 2017 Dec 13;7:17500. doi: 10.1038/s41598-017-17556-y (PMC5727508; doi:10.1038/s41598-017-17556-y)
Supplement: Supplementary file 1 — Supplementary Information [file 41598_2017_17556_MOESM1_ESM.pdf]

The development of spontaneous facial responses to others' emotions in infancy. An EMG study.

Jakob Kaiser, Maria Magdalena Crespo-Llado, Chiara Turati, and Elena Geangu

Supplementary Information

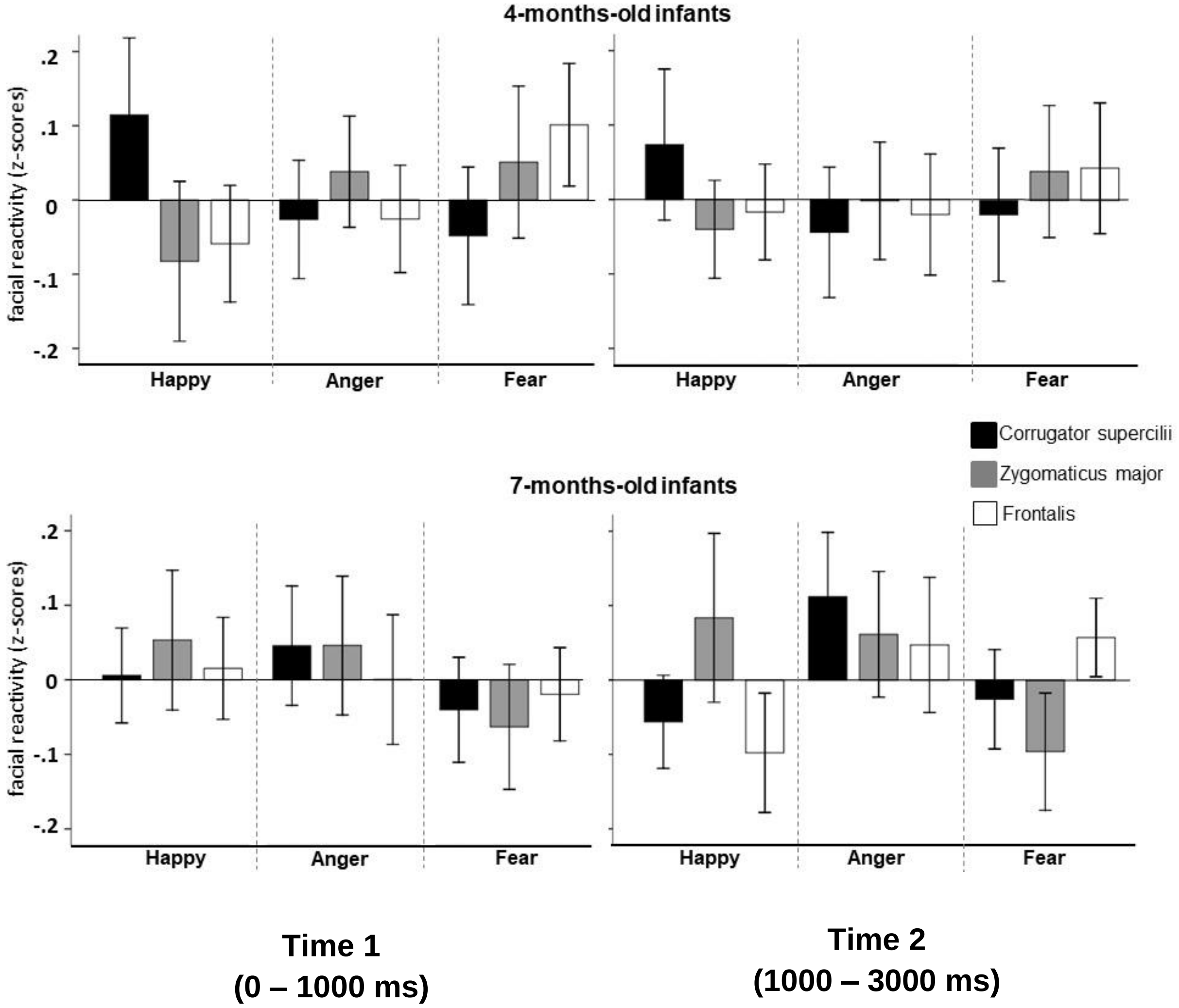

Supplementary Figure 1. Means (and 95% confidence interval) of facial reactions towards the stimuli during Time 1 (0-1000ms from onset) and Time 2 (1000-3000ms from onset) for different muscles (expressed as z-scores).

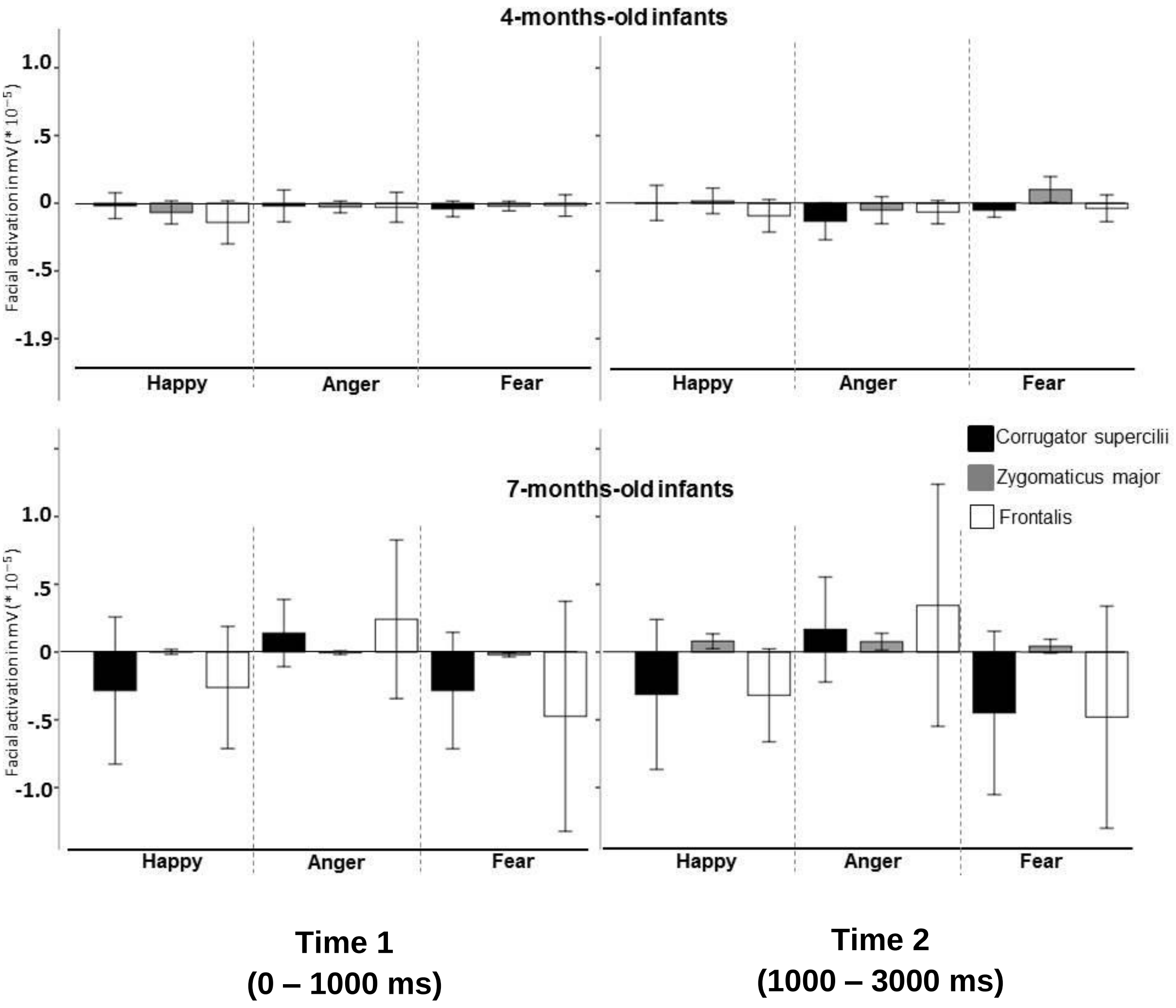

Supplementary Figure 2. Means (and 95% confidence interval) of facial reactions towards the stimuli before data standardisations. Analysis of variance analogous to main analysis indicates no significant effects of time, muscle, emotion, or age (all p's > .05).
